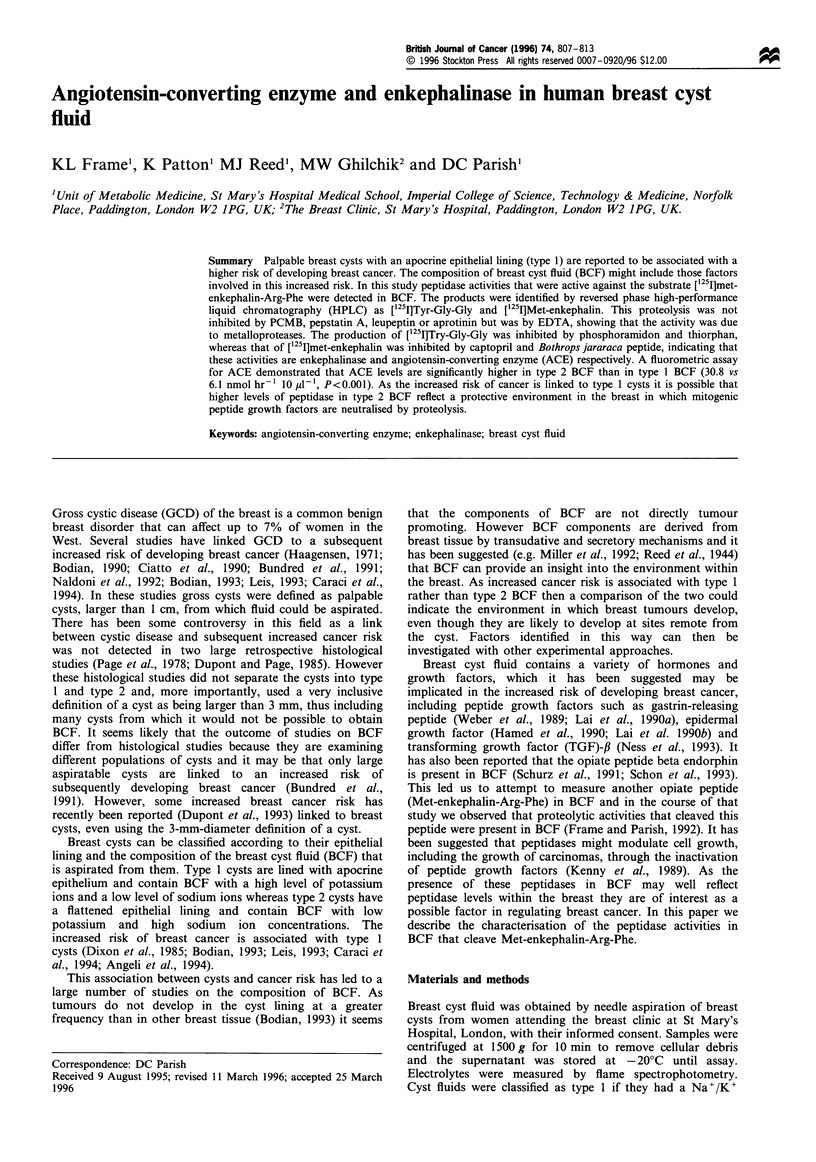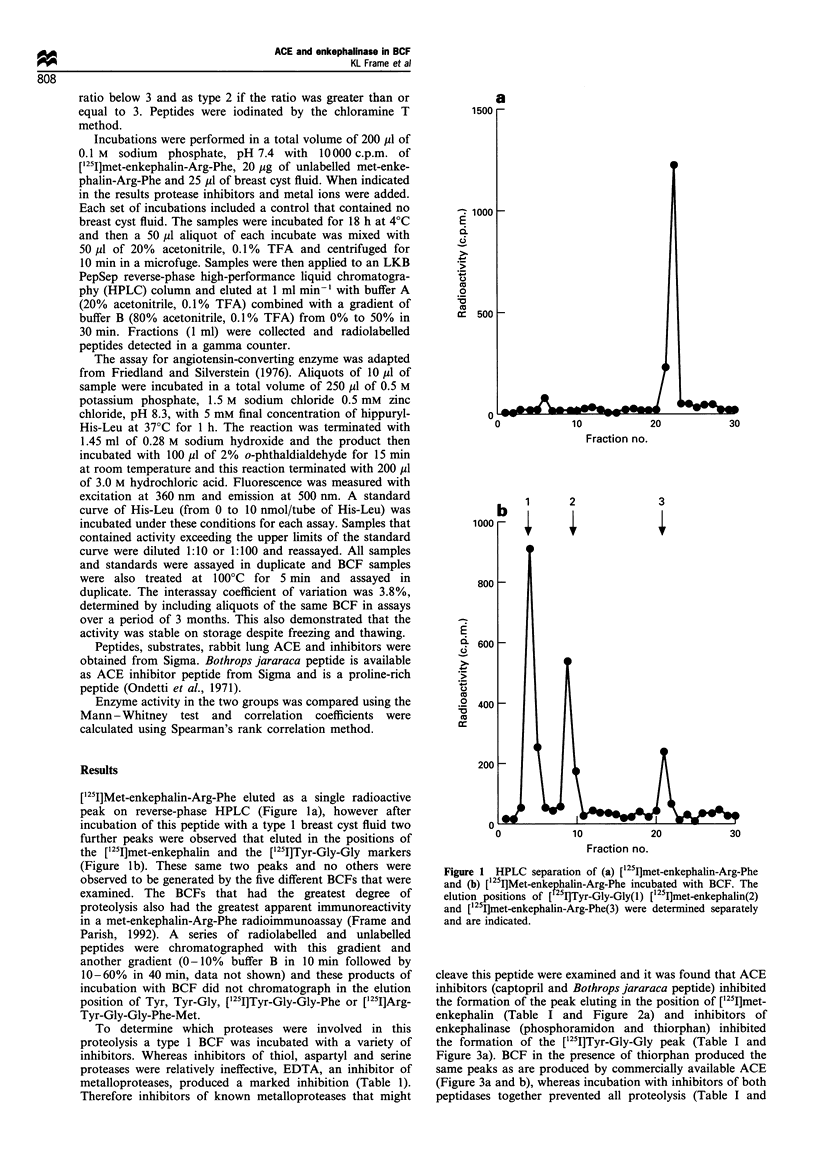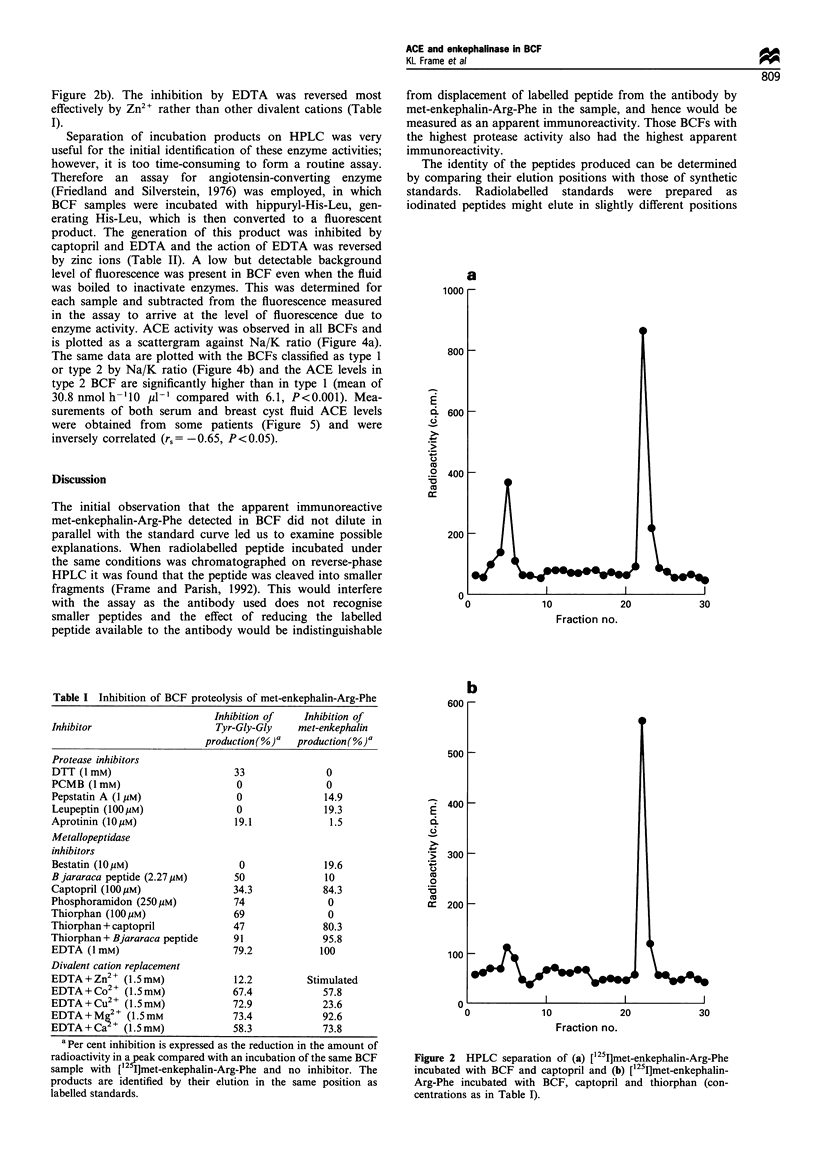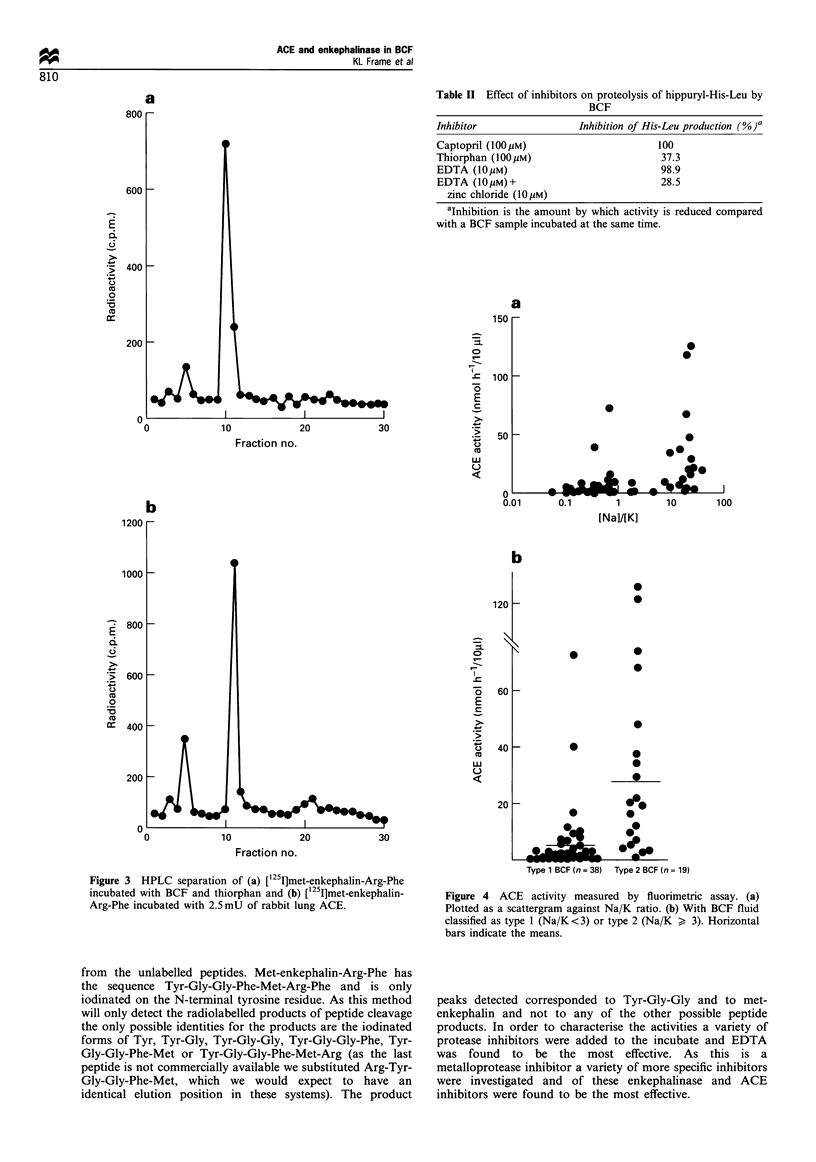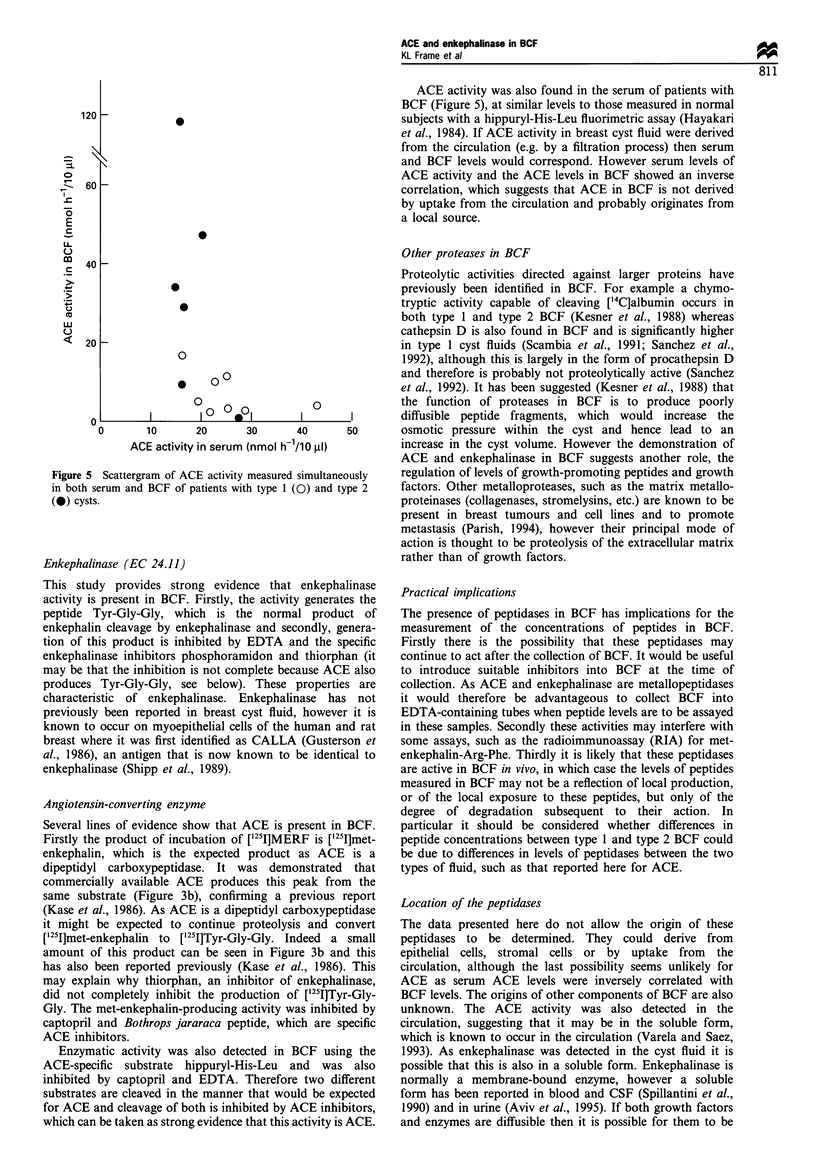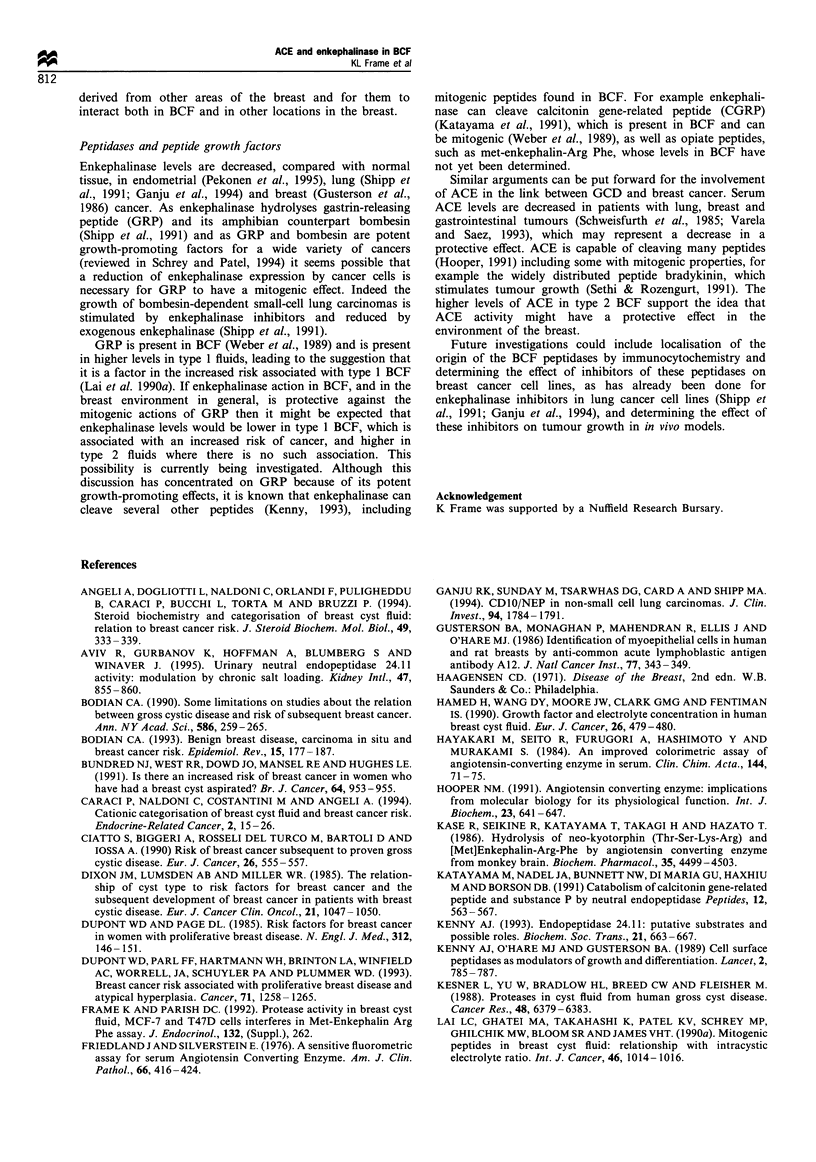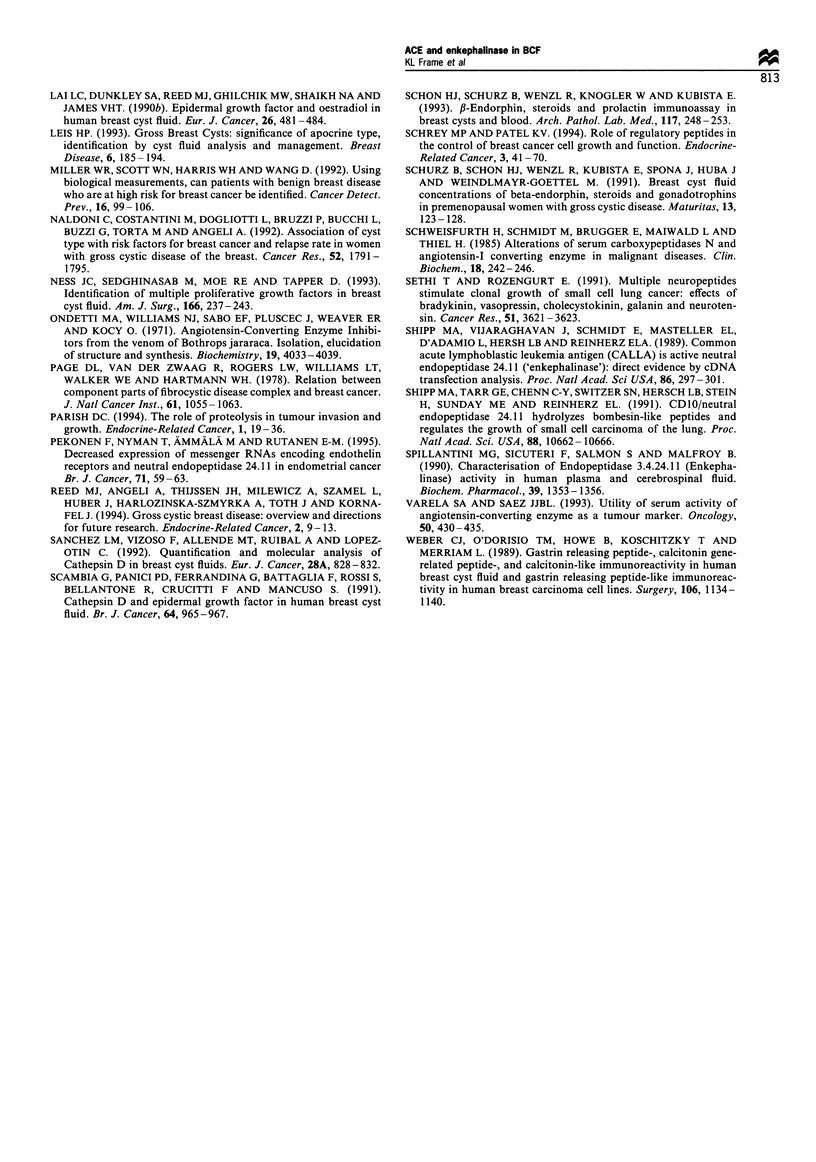# Angiotensin-converting enzyme and enkephalinase in human breast cyst fluid.

**DOI:** 10.1038/bjc.1996.440

**Published:** 1996-09

**Authors:** K. L. Frame, K. Patton, M. J. Reed, M. W. Ghilchik, D. C. Parish

**Affiliations:** Unit of Metabolic Medicine, St Mary's Hospital Medical School, Imperial College of Science, Technology & Medicine, London, UK.

## Abstract

Palpable breast cysts with an apocrine epithelial lining (type 1) are reported to be associated with a higher risk of developing breast cancer. The composition of breast cyst fluid (BCF) might include those factors involved in this increased risk. In this study peptidase activities that were active against the substrate [125I]metenkephalin-Arg-Phe were detected in BCF. The products were identified by reversed phase high-performance liquid chromatography (HPLC) as [125I]Tyr-Gly-Gly and [125I]Met-enkephalin. This proteolysis was not inhibited by PCMB, pepstatin A, leupeptin or aprotinin but was by EDTA, showing that the activity was due to metalloproteases. The production of [125I]Try-Gly-Gly was inhibited by phosphoramidon and thiorphan, whereas that of [125I]met-enkephalin was inhibited by captopril and Bothrops jararaca peptide, indicating that these activities are enkephalinase and angiotensin-converting enzyme (ACE) respectively. A fluorometric assay for ACE demonstrated that ACE levels are significantly higher in type 2 BCF than in type 1 BCF (30.8 vs 6.1 nmol hr-1 10 microliters-1, P < 0.001). As the increased risk of cancer is linked to type 1 cysts it is possible that higher levels of peptidase in type 2 BCF reflect a protective environment in the breast in which mitogenic peptide growth factors are neutralised by proteolysis.


British Journal of Cancer (1996) 74, 807-813
© 1996 Stockton Press All rights reserved 0007-0920/96 $12.00

### Angiotensin-converting enzyme and enkephalinase in human breast cyst fluid

KL Frame[1], K Patton[1] MJ Reed[1], MW Ghilchik[2] and DC Parish[1]

[1]*Unit of Metabolic Medicine, St Mary's Hospital Medical School, Imperial College of Science, Technology & Medicine, Norfolk Place, Paddington, London W2 1PG, UK;* [2]*The Breast Clinic, St Mary's Hospital, Paddington, London W2 1PG, UK.*

**Summary** Palpable breast cysts with an apocrine epithelial lining (type 1) are reported to be associated with a higher risk of developing breast cancer. The composition of breast cyst fluid (BCF) might include those factors involved in this increased risk. In this study peptidase activities that were active against the substrate [$^{125}$I]met-enkephalin-Arg-Phe were detected in BCF. The products were identified by reversed phase high-performance liquid chromatography (HPLC) as [$^{125}$I]Tyr-Gly-Gly and [$^{125}$I]Met-enkephalin. This proteolysis was not inhibited by PCMB, pepstatin A, leupeptin or aprotinin but was by EDTA, showing that the activity was due to metalloproteases. The production of [$^{125}$I]Try-Gly-Gly was inhibited by phosphoramidon and thiorphan, whereas that of [$^{125}$I]met-enkephalin was inhibited by captopril and *Bothrops jararaca* peptide, indicating that these activities are enkephalinase and angiotensin-converting enzyme (ACE) respectively. A fluorometric assay for ACE demonstrated that ACE levels are significantly higher in type 2 BCF than in type 1 BCF (30.8 *vs* 6.1 nmol $hr^{-1}$ 10 $\mu l^{-1}$, $P<0.001$). As the increased risk of cancer is linked to type 1 cysts it is possible that higher levels of peptidase in type 2 BCF reflect a protective environment in the breast in which mitogenic peptide growth factors are neutralised by proteolysis.

**Keywords:** angiotensin-converting enzyme; enkephalinase; breast cyst fluid

Gross cystic disease (GCD) of the breast is a common benign breast disorder that can affect up to 7% of women in the West. Several studies have linked GCD to a subsequent increased risk of developing breast cancer (Haagensen, 1971; Bodian, 1990; Ciatto *et al.*, 1990; Bundred *et al.*, 1991; Naldoni *et al.*, 1992; Bodian, 1993; Leis, 1993; Caraci *et al.*, 1994). In these studies gross cysts were defined as palpable cysts, larger than 1 cm, from which fluid could be aspirated. There has been some controversy in this field as a link between cystic disease and subsequent increased cancer risk was not detected in two large retrospective histological studies (Page *et al.*, 1978; Dupont and Page, 1985). However these histological studies did not separate the cysts into type 1 and type 2 and, more importantly, used a very inclusive definition of a cyst as being larger than 3 mm, thus including many cysts from which it would not be possible to obtain BCF. It seems likely that the outcome of studies on BCF differ from histological studies because they are examining different populations of cysts and it may be that only large aspiratable cysts are linked to an increased risk of subsequently developing breast cancer (Bundred *et al.*, 1991). However, some increased breast cancer risk has recently been reported (Dupont *et al.*, 1993) linked to breast cysts, even using the 3-mm-diameter definition of a cyst.

Breast cysts can be classified according to their epithelial lining and the composition of the breast cyst fluid (BCF) that is aspirated from them. Type 1 cysts are lined with apocrine epithelium and contain BCF with a high level of potassium ions and a low level of sodium ions whereas type 2 cysts have a flattened epithelial lining and contain BCF with low potassium and high sodium ion concentrations. The increased risk of breast cancer is associated with type 1 cysts (Dixon *et al.*, 1985; Bodian, 1993; Leis, 1993; Caraci *et al.*, 1994; Angeli *et al.*, 1994).

This association between cysts and cancer risk has led to a large number of studies on the composition of BCF. As tumours do not develop in the cyst lining at a greater frequency than in other breast tissue (Bodian, 1993) it seems that the components of BCF are not directly tumour promoting. However BCF components are derived from breast tissue by transudative and secretory mechanisms and it has been suggested (e.g. Miller *et al.*, 1992; Reed *et al.*, 1944) that BCF can provide an insight into the environment within the breast. As increased cancer risk is associated with type 1 rather than type 2 BCF then a comparison of the two could indicate the environment in which breast tumours develop, even though they are likely to develop at sites remote from the cyst. Factors identified in this way can then be investigated with other experimental approaches.

Breast cyst fluid contains a variety of hormones and growth factors, which it has been suggested may be implicated in the increased risk of developing breast cancer, including peptide growth factors such as gastrin-releasing peptide (Weber *et al.*, 1989; Lai *et al.*, 1990*a*), epidermal growth factor (Hamed *et al.*, 1990; Lai *et al.* 1990*b*) and transforming growth factor (TGF)-$\beta$ (Ness *et al.*, 1993). It has also been reported that the opiate peptide beta endorphin is present in BCF (Schurz *et al.*, 1991; Schon *et al.*, 1993). This led us to attempt to measure another opiate peptide (Met-enkephalin-Arg-Phe) in BCF and in the course of that study we observed that proteolytic activities that cleaved this peptide were present in BCF (Frame and Parish, 1992). It has been suggested that peptidases might modulate cell growth, including the growth of carcinomas, through the inactivation of peptide growth factors (Kenny *et al.*, 1989). As the presence of these peptidases in BCF may well reflect peptidase levels within the breast they are of interest as a possible factor in regulating breast cancer. In this paper we describe the characterisation of the peptidase activities in BCF that cleave Met-enkephalin-Arg-Phe.

### Materials and methods

Breast cyst fluid was obtained by needle aspiration of breast cysts from women attending the breast clinic at St Mary's Hospital, London, with their informed consent. Samples were centrifuged at 1500 *g* for 10 min to remove cellular debris and the supernatant was stored at −20°C until assay. Electrolytes were measured by flame spectrophotometry. Cyst fluids were classified as type 1 if they had a $Na^+/K^+$

Correspondence: DC Parish
Received 9 August 1995; revised 11 March 1996; accepted 25 March 1996

ratio below 3 and as type 2 if the ratio was greater than or equal to 3. Peptides were iodinated by the chloramine T method.

Incubations were performed in a total volume of 200 μl of 0.1 M sodium phosphate, pH 7.4 with 10 000 c.p.m. of [$^{125}$I]met-enkephalin-Arg-Phe, 20 μg of unlabelled met-enkephalin-Arg-Phe and 25 μl of breast cyst fluid. When indicated in the results protease inhibitors and metal ions were added. Each set of incubations included a control that contained no breast cyst fluid. The samples were incubated for 18 h at 4°C and then a 50 μl aliquot of each incubate was mixed with 50 μl of 20% acetonitrile, 0.1% TFA and centrifuged for 10 min in a microfuge. Samples were then applied to an LKB PepSep reverse-phase high-performance liquid chromatography (HPLC) column and eluted at 1 ml min$^{-1}$ with buffer A (20% acetonitrile, 0.1% TFA) combined with a gradient of buffer B (80% acetonitrile, 0.1% TFA) from 0% to 50% in 30 min. Fractions (1 ml) were collected and radiolabelled peptides detected in a gamma counter.

The assay for angiotensin-converting enzyme was adapted from Friedland and Silverstein (1976). Aliquots of 10 μl of sample were incubated in a total volume of 250 μl of 0.5 M potassium phosphate, 1.5 M sodium chloride 0.5 mM zinc chloride, pH 8.3, with 5 mM final concentration of hippuryl-His-Leu at 37°C for 1 h. The reaction was terminated with 1.45 ml of 0.28 M sodium hydroxide and the product then incubated with 100 μl of 2% *o*-phthaldialdehyde for 15 min at room temperature and this reaction terminated with 200 μl of 3.0 M hydrochloric acid. Fluorescence was measured with excitation at 360 nm and emission at 500 nm. A standard curve of His-Leu (from 0 to 10 nmol/tube of His-Leu) was incubated under these conditions for each assay. Samples that contained activity exceeding the upper limits of the standard curve were diluted 1:10 or 1:100 and reassayed. All samples and standards were assayed in duplicate and BCF samples were also treated at 100°C for 5 min and assayed in duplicate. The interassay coefficient of variation was 3.8%, determined by including aliquots of the same BCF in assays over a period of 3 months. This also demonstrated that the activity was stable on storage despite freezing and thawing.

Peptides, substrates, rabbit lung ACE and inhibitors were obtained from Sigma. *Bothrops jararaca* peptide is available as ACE inhibitor peptide from Sigma and is a proline-rich peptide (Ondetti *et al.*, 1971).

Enzyme activity in the two groups was compared using the Mann–Whitney test and correlation coefficients were calculated using Spearman's rank correlation method.

### Results

[$^{125}$I]Met-enkephalin-Arg-Phe eluted as a single radioactive peak on reverse-phase HPLC (Figure 1a), however after incubation of this peptide with a type 1 breast cyst fluid two further peaks were observed that eluted in the positions of the [$^{125}$I]met-enkephalin and the [$^{125}$I]Tyr-Gly-Gly markers (Figure 1b). These same two peaks and no others were observed to be generated by the five different BCFs that were examined. The BCFs that had the greatest degree of proteolysis also had the greatest apparent immunoreactivity in a met-enkephalin-Arg-Phe radioimmunoassay (Frame and Parish, 1992). A series of radiolabelled and unlabelled peptides were chromatographed with this gradient and another gradient (0–10% buffer B in 10 min followed by 10–60% in 40 min, data not shown) and these products of incubation with BCF did not chromatograph in the elution position of Tyr, Tyr-Gly, [$^{125}$I]Tyr-Gly-Gly-Phe or [$^{125}$I]Arg-Tyr-Gly-Gly-Phe-Met.

To determine which proteases were involved in this proteolysis a type 1 BCF was incubated with a variety of inhibitors. Whereas inhibitors of thiol, aspartyl and serine proteases were relatively ineffective, EDTA, an inhibitor of metalloproteases, produced a marked inhibition (Table 1). Therefore inhibitors of known metalloproteases that might

**Figure 1** HPLC separation of (**a**) [$^{125}$I]met-enkephalin-Arg-Phe and (**b**) [$^{125}$I]Met-enkephalin-Arg-Phe incubated with BCF. The elution positions of [$^{125}$I]Tyr-Gly-Gly(1) [$^{125}$I]met-enkephalin(2) and [$^{125}$I]met-enkephalin-Arg-Phe(3) were determined separately and are indicated.

cleave this peptide were examined and it was found that ACE inhibitors (captopril and *Bothrops jararaca* peptide) inhibited the formation of the peak eluting in the position of [$^{125}$I]met-enkephalin (Table I and Figure 2a) and inhibitors of enkephalinase (phosphoramidon and thiorphan) inhibited the formation of the [$^{125}$I]Tyr-Gly-Gly peak (Table I and Figure 3a). BCF in the presence of thiorphan produced the same peaks as are produced by commercially available ACE (Figure 3a and b), whereas incubation with inhibitors of both peptidases together prevented all proteolysis (Table I and

Figure 2b). The inhibition by EDTA was reversed most effectively by $Zn^{2+}$ rather than other divalent cations (Table I).

Separation of incubation products on HPLC was very useful for the initial identification of these enzyme activities; however, it is too time-consuming to form a routine assay. Therefore an assay for angiotensin-converting enzyme (Friedland and Silverstein, 1976) was employed, in which BCF samples were incubated with hippuryl-His-Leu, generating His-Leu, which is then converted to a fluorescent product. The generation of this product was inhibited by captopril and EDTA and the action of EDTA was reversed by zinc ions (Table II). A low but detectable background level of fluorescence was present in BCF even when the fluid was boiled to inactivate enzymes. This was determined for each sample and subtracted from the fluorescence measured in the assay to arrive at the level of fluorescence due to enzyme activity. ACE activity was observed in all BCFs and is plotted as a scattergram against Na/K ratio (Figure 4a). The same data are plotted with the BCFs classified as type 1 or type 2 by Na/K ratio (Figure 4b) and the ACE levels in type 2 BCF are significantly higher than in type 1 (mean of 30.8 nmol $h^{-1}$10 $\mu l^{-1}$ compared with 6.1, $P<0.001$). Measurements of both serum and breast cyst fluid ACE levels were obtained from some patients (Figure 5) and were inversely correlated ($r_s = -0.65$, $P<0.05$).

### Discussion

The initial observation that the apparent immunoreactive met-enkephalin-Arg-Phe detected in BCF did not dilute in parallel with the standard curve led us to examine possible explanations. When radiolabelled peptide incubated under the same conditions was chromatographed on reverse-phase HPLC it was found that the peptide was cleaved into smaller fragments (Frame and Parish, 1992). This would interfere with the assay as the antibody used does not recognise smaller peptides and the effect of reducing the labelled peptide available to the antibody would be indistinguishable from displacement of labelled peptide from the antibody by met-enkephalin-Arg-Phe in the sample, and hence would be measured as an apparent immunoreactivity. Those BCFs with the highest protease activity also had the highest apparent immunoreactivity.

The identity of the peptides produced can be determined by comparing their elution positions with those of synthetic standards. Radiolabelled standards were prepared as iodinated peptides might elute in slightly different positions

**Table I** Inhibition of BCF proteolysis of met-enkephalin-Arg-Phe

| *Inhibitor* | *Inhibition of Tyr-Gly-Gly production(%)*[a] | *Inhibition of met-enkephalin production(%)*[a] |
|---|---|---|
| *Protease inhibitors* | | |
| DTT (1 mM) | 33 | 0 |
| PCMB (1 mM) | 0 | 0 |
| Pepstatin A (1 μM) | 0 | 14.9 |
| Leupeptin (100 μM) | 0 | 19.3 |
| Aprotinin (10 μM) | 19.1 | 1.5 |
| *Metallopeptidase inhibitors* | | |
| Bestatin (10 μM) | 0 | 19.6 |
| *B jararaca* peptide (2.27 μM) | 50 | 10 |
| Captopril (100 μM) | 34.3 | 84.3 |
| Phosphoramidon (250 μM) | 74 | 0 |
| Thiorphan (100 μM) | 69 | 0 |
| Thiorphan + captopril | 47 | 80.3 |
| Thiorphan + *B jararaca* peptide | 91 | 95.8 |
| EDTA (1 mM) | 79.2 | 100 |
| *Divalent cation replacement* | | |
| EDTA + $Zn^{2+}$ (1.5 mM) | 12.2 | Stimulated |
| EDTA + $Co^{2+}$ (1.5 mM) | 67.4 | 57.8 |
| EDTA + $Cu^{2+}$ (1.5 mM) | 72.9 | 23.6 |
| EDTA + $Mg^{2+}$ (1.5 mM | 73.4 | 92.6 |
| EDTA + $Ca^{2+}$ (1.5 mM) | 58.3 | 73.8 |

[a] Per cent inhibition is expressed as the reduction in the amount of radioactivity in a peak compared with an incubation of the same BCF sample with [$^{125}$I]met-enkephalin-Arg-Phe and no inhibitor. The products are identified by their elution in the same position as labelled standards.

**Figure 2** HPLC separation of (**a**) [$^{125}$I]met-enkephalin-Arg-Phe incubated with BCF and captopril and (**b**) [$^{125}$I]met-enkephalin-Arg-Phe incubated with BCF, captopril and thiorphan (concentrations as in Table I).

**Figure 3** HPLC separation of (**a**) [$^{125}$I]met-enkephalin-Arg-Phe incubated with BCF and thiorphan and (**b**) [$^{125}$I]met-enkephalin-Arg-Phe incubated with 2.5 mU of rabbit lung ACE.

**Table II** Effect of inhibitors on proteolysis of hippuryl-His-Leu by BCF

| *Inhibitor* | *Inhibition of His-Leu production (%)*[a] |
|---|---|
| Captopril (100 μM) | 100 |
| Thiorphan (100 μM) | 37.3 |
| EDTA (10 μM) | 98.9 |
| EDTA (10 μM) + zinc chloride (10 μM) | 28.5 |

[a]Inhibition is the amount by which activity is reduced compared with a BCF sample incubated at the same time.

**Figure 4** ACE activity measured by fluorimetric assay. (**a**) Plotted as a scattergram against Na/K ratio. (**b**) With BCF fluid classified as type 1 (Na/K < 3) or type 2 (Na/K ⩾ 3). Horizontal bars indicate the means.

from the unlabelled peptides. Met-enkephalin-Arg-Phe has the sequence Tyr-Gly-Gly-Phe-Met-Arg-Phe and is only iodinated on the N-terminal tyrosine residue. As this method will only detect the radiolabelled products of peptide cleavage the only possible identities for the products are the iodinated forms of Tyr, Tyr-Gly, Tyr-Gly-Gly, Tyr-Gly-Gly-Phe, Tyr-Gly-Gly-Phe-Met or Tyr-Gly-Gly-Phe-Met-Arg (as the last peptide is not commercially available we substituted Arg-Tyr-Gly-Gly-Phe-Met, which we would expect to have an identical elution position in these systems). The product peaks detected corresponded to Tyr-Gly-Gly and to met-enkephalin and not to any of the other possible peptide products. In order to characterise the activities a variety of protease inhibitors were added to the incubate and EDTA was found to be the most effective. As this is a metalloprotease inhibitor a variety of more specific inhibitors were investigated and of these enkephalinase and ACE inhibitors were found to be the most effective.

**Figure 5** Scattergram of ACE activity measured simultaneously in both serum and BCF of patients with type 1 (○) and type 2 (●) cysts.

*Enkephalinase (EC 24.11)*

This study provides strong evidence that enkephalinase activity is present in BCF. Firstly, the activity generates the peptide Tyr-Gly-Gly, which is the normal product of enkephalin cleavage by enkephalinase and secondly, generation of this product is inhibited by EDTA and the specific enkephalinase inhibitors phosphoramidon and thiorphan (it may be that the inhibition is not complete because ACE also produces Tyr-Gly-Gly, see below). These properties are characteristic of enkephalinase. Enkephalinase has not previously been reported in breast cyst fluid, however it is known to occur on myoepithelial cells of the human and rat breast where it was first identified as CALLA (Gusterson *et al.*, 1986), an antigen that is now known to be identical to enkephalinase (Shipp *et al.*, 1989).

*Angiotensin-converting enzyme*

Several lines of evidence show that ACE is present in BCF. Firstly the product of incubation of [$^{125}$I]MERF is [$^{125}$I]met-enkephalin, which is the expected product as ACE is a dipeptidyl carboxypeptidase. It was demonstrated that commercially available ACE produces this peak from the same substrate (Figure 3b), confirming a previous report (Kase *et al.*, 1986). As ACE is a dipeptidyl carboxypeptidase it might be expected to continue proteolysis and convert [$^{125}$I]met-enkephalin to [$^{125}$I]Tyr-Gly-Gly. Indeed a small amount of this product can be seen in Figure 3b and this has also been reported previously (Kase *et al.*, 1986). This may explain why thiorphan, an inhibitor of enkephalinase, did not completely inhibit the production of [$^{125}$I]Tyr-Gly-Gly. The met-enkephalin-producing activity was inhibited by captopril and *Bothrops jararaca* peptide, which are specific ACE inhibitors.

Enzymatic activity was also detected in BCF using the ACE-specific substrate hippuryl-His-Leu and was also inhibited by captopril and EDTA. Therefore two different substrates are cleaved in the manner that would be expected for ACE and cleavage of both is inhibited by ACE inhibitors, which can be taken as strong evidence that this activity is ACE.

ACE activity was also found in the serum of patients with BCF (Figure 5), at similar levels to those measured in normal subjects with a hippuryl-His-Leu fluorimetric assay (Hayakari *et al.*, 1984). If ACE activity in breast cyst fluid were derived from the circulation (e.g. by a filtration process) then serum and BCF levels would correspond. However serum levels of ACE activity and the ACE levels in BCF showed an inverse correlation, which suggests that ACE in BCF is not derived by uptake from the circulation and probably originates from a local source.

*Other proteases in BCF*

Proteolytic activities directed against larger proteins have previously been identified in BCF. For example a chymotryptic activity capable of cleaving [$^{14}$C]albumin occurs in both type 1 and type 2 BCF (Kesner *et al.*, 1988) whereas cathepsin D is also found in BCF and is significantly higher in type 1 cyst fluids (Scambia *et al.*, 1991; Sanchez *et al.*, 1992), although this is largely in the form of procathepsin D and therefore is probably not proteolytically active (Sanchez *et al.*, 1992). It has been suggested (Kesner *et al.*, 1988) that the function of proteases in BCF is to produce poorly diffusible peptide fragments, which would increase the osmotic pressure within the cyst and hence lead to an increase in the cyst volume. However the demonstration of ACE and enkephalinase in BCF suggests another role, the regulation of levels of growth-promoting peptides and growth factors. Other metalloproteases, such as the matrix metalloproteinases (collagenases, stromelysins, etc.) are known to be present in breast tumours and cell lines and to promote metastasis (Parish, 1994), however their principal mode of action is thought to be proteolysis of the extracellular matrix rather than of growth factors.

*Practical implications*

The presence of peptidases in BCF has implications for the measurement of the concentrations of peptides in BCF. Firstly there is the possibility that these peptidases may continue to act after the collection of BCF. It would be useful to introduce suitable inhibitors into BCF at the time of collection. As ACE and enkephalinase are metallopeptidases it would therefore be advantageous to collect BCF into EDTA-containing tubes when peptide levels are to be assayed in these samples. Secondly these activities may interfere with some assays, such as the radioimmunoassay (RIA) for met-enkephalin-Arg-Phe. Thirdly it is likely that these peptidases are active in BCF *in vivo*, in which case the levels of peptides measured in BCF may not be a reflection of local production, or of the local exposure to these peptides, but only of the degree of degradation subsequent to their action. In particular it should be considered whether differences in peptide concentrations between type 1 and type 2 BCF could be due to differences in levels of peptidases between the two types of fluid, such as that reported here for ACE.

*Location of the peptidases*

The data presented here do not allow the origin of these peptidases to be determined. They could derive from epithelial cells, stromal cells or by uptake from the circulation, although the last possibility seems unlikely for ACE as serum ACE levels were inversely correlated with BCF levels. The origins of other components of BCF are also unknown. The ACE activity was also detected in the circulation, suggesting that it may be in the soluble form, which is known to occur in the circulation (Varela and Saez, 1993). As enkephalinase was detected in the cyst fluid it is possible that this is also in a soluble form. Enkephalinase is normally a membrane-bound enzyme, however a soluble form has been reported in blood and CSF (Spillantini *et al.*, 1990) and in urine (Aviv *et al.*, 1995). If both growth factors and enzymes are diffusible then it is possible for them to be derived from other areas of the breast and for them to interact both in BCF and in other locations in the breast.

### *Peptidases and peptide growth factors*

Enkephalinase levels are decreased, compared with normal tissue, in endometrial (Pekonen *et al.*, 1995), lung (Shipp *et al.*, 1991; Ganju *et al.*, 1994) and breast (Gusterson *et al.*, 1986) cancer. As enkephalinase hydrolyses gastrin-releasing peptide (GRP) and its amphibian counterpart bombesin (Shipp *et al.*, 1991) and as GRP and bombesin are potent growth-promoting factors for a wide variety of cancers (reviewed in Schrey and Patel, 1994) it seems possible that a reduction of enkephalinase expression by cancer cells is necessary for GRP to have a mitogenic effect. Indeed the growth of bombesin-dependent small-cell lung carcinomas is stimulated by enkephalinase inhibitors and reduced by exogenous enkephalinase (Shipp *et al.*, 1991).

GRP is present in BCF (Weber *et al.*, 1989) and is present in higher levels in type 1 fluids, leading to the suggestion that it is a factor in the increased risk associated with type 1 BCF (Lai *et al.* 1990*a*). If enkephalinase action in BCF, and in the breast environment in general, is protective against the mitogenic actions of GRP then it might be expected that enkephalinase levels would be lower in type 1 BCF, which is associated with an increased risk of cancer, and higher in type 2 fluids where there is no such association. This possibility is currently being investigated. Although this discussion has concentrated on GRP because of its potent growth-promoting effects, it is known that enkephalinase can cleave several other peptides (Kenny, 1993), including mitogenic peptides found in BCF. For example enkephalinase can cleave calcitonin gene-related peptide (CGRP) (Katayama *et al.*, 1991), which is present in BCF and can be mitogenic (Weber *et al.*, 1989), as well as opiate peptides, such as met-enkephalin-Arg Phe, whose levels in BCF have not yet been determined.

Similar arguments can be put forward for the involvement of ACE in the link between GCD and breast cancer. Serum ACE levels are decreased in patients with lung, breast and gastrointestinal tumours (Schweisfurth *et al.*, 1985; Varela and Saez, 1993), which may represent a decrease in a protective effect. ACE is capable of cleaving many peptides (Hooper, 1991) including some with mitogenic properties, for example the widely distributed peptide bradykinin, which stimulates tumour growth (Sethi & Rozengurt, 1991). The higher levels of ACE in type 2 BCF support the idea that ACE activity might have a protective effect in the environment of the breast.

Future investigations could include localisation of the origin of the BCF peptidases by immunocytochemistry and determining the effect of inhibitors of these peptidases on breast cancer cell lines, as has already been done for enkephalinase inhibitors in lung cancer cell lines (Shipp *et al.*, 1991; Ganju *et al.*, 1994), and determining the effect of these inhibitors on tumour growth in *in vivo* models.

**Acknowledgement**

K Frame was supported by a Nuffield Research Bursary.

### References

ANGELI A, DOGLIOTTI L, NALDONI C, ORLANDI F, PULIGHEDDU B, CARACI P, BUCCHI L, TORTA M AND BRUZZI P. (1994). Steroid biochemistry and categorisation of breast cyst fluid: relation to breast cancer risk. *J. Steroid Biochem. Mol. Biol.*, **49**, 333–339.

AVIV R, GURBANOV K, HOFFMAN A, BLUMBERG S AND WINAVER J. (1995). Urinary neutral endopeptidase 24.11 activity: modulation by chronic salt loading. *Kidney Intl.*, **47**, 855–860.

BODIAN CA. (1990). Some limitations on studies about the relation between gross cystic disease and risk of subsequent breast cancer. *Ann. NY Acad. Sci.*, **586**, 259–265.

BODIAN CA. (1993). Benign breast disease, carcinoma in situ and breast cancer risk. *Epidemiol. Rev.*, **15**, 177–187.

BUNDRED NJ, WEST RR, DOWD JO, MANSEL RE AND HUGHES LE. (1991). Is there an increased risk of breast cancer in women who have had a breast cyst aspirated? *Br. J. Cancer*, **64**, 953–955.

CARACI P, NALDONI C, COSTANTINI M AND ANGELI A. (1994). Cationic categorisation of breast cyst fluid and breast cancer risk. *Endocrine-Related Cancer*, **2**, 15–26.

CIATTO S, BIGGERI A, ROSSELI DEL TURCO M, BARTOLI D AND IOSSA A. (1990) Risk of breast cancer subsequent to proven gross cystic disease. *Eur. J. Cancer*, **26**, 555–557.

DIXON JM, LUMSDEN AB AND MILLER WR. (1985). The relationship of cyst type to risk factors for breast cancer and the subsequent development of breast cancer in patients with breast cystic disease. *Eur. J. Cancer Clin. Oncol.*, **21**, 1047–1050.

DUPONT WD AND PAGE DL. (1985). Risk factors for breast cancer in women with proliferative breast disease. *N. Engl. J. Med.*, **312**, 146–151.

DUPONT WD, PARL FF, HARTMANN WH, BRINTON LA, WINFIELD AC, WORRELL, JA, SCHUYLER PA AND PLUMMER WD. (1993). Breast cancer risk associated with proliferative breast disease and atypical hyperplasia. *Cancer*, **71**, 1258–1265.

FRAME K AND PARISH DC. (1992). Protease activity in breast cyst fluid, MCF-7 and T47D cells interferes in Met-Enkephalin Arg Phe assay. *J. Endocrinol.*, **132**, (Suppl.), 262.

FRIEDLAND J AND SILVERSTEIN E. (1976). A sensitive fluorometric assay for serum Angiotensin Converting Enzyme. *Am. J. Clin. Pathol.*, **66**, 416–424.

GANJU RK, SUNDAY M, TSARWHAS DG, CARD A AND SHIPP MA. (1994). CD10/NEP in non-small cell lung carcinomas. *J. Clin. Invest.*, **94**, 1784–1791.

GUSTERSON BA, MONAGHAN P, MAHENDRAN R, ELLIS J AND O'HARE MJ. (1986) Identification of myoepithelial cells in human and rat breasts by anti-common acute lymphoblastic antigen antibody A12. *J. Natl Cancer Inst.*, **77**, 343–349.

HAAGENSEN CD. (1971). *Disease of the Breast*, 2nd edn. W.B. Saunders & Co.: Philadelphia.

HAMED H, WANG DY, MOORE JW, CLARK GMG AND FENTIMAN IS. (1990). Growth factor and electrolyte concentration in human breast cyst fluid. *Eur. J. Cancer*, **26**, 479–480.

HAYAKARI M, SEITO R, FURUGORI A, HASHIMOTO Y AND MURAKAMI S. (1984). An improved colorimetric assay of angiotensin-converting enzyme in serum. *Clin. Chim. Acta.*, **144**, 71–75.

HOOPER NM. (1991). Angiotensin converting enzyme: implications from molecular biology for its physiological function. *Int. J. Biochem.*, **23**, 641–647.

KASE R, SEIKINE R, KATAYAMA T, TAKAGI H AND HAZATO T. (1986). Hydrolysis of neo-kyotorphin (Thr-Ser-Lys-Arg) and [Met]Enkephalin-Arg-Phe by angiotensin converting enzyme from monkey brain. *Biochem. Pharmacol.*, **35**, 4499–4503.

KATAYAMA M, NADEL JA, BUNNETT NW, DI MARIA GU, HAXHIU M AND BORSON DB. (1991) Catabolism of calcitonin gene-related peptide and substance P by neutral endopeptidase *Peptides*, **12**, 563–567.

KENNY AJ. (1993). Endopeptidase 24.11: putative substrates and possible roles. *Biochem. Soc. Trans.*, **21**, 663–667.

KENNY AJ, O'HARE MJ AND GUSTERSON BA. (1989) Cell surface peptidases as modulators of growth and differentiation. *Lancet*, **2**, 785–787.

KESNER L, YU W, BRADLOW HL, BREED CW AND FLEISHER M. (1988). Proteases in cyst fluid from human gross cyst disease. *Cancer Res.*, **48**, 6379–6383.

LAI LC, GHATEI MA, TAKAHASHI K, PATEL KV, SCHREY MP, GHILCHIK MW, BLOOM SR AND JAMES VHT. (1990*a*). Mitogenic peptides in breast cyst fluid: relationship with intracystic electrolyte ratio. *Int. J. Cancer*, **46**, 1014–1016.

LAI LC, DUNKLEY SA, REED MJ, GHILCHIK MW, SHAIKH NA AND JAMES VHT. (1990*b*). Epidermal growth factor and oestradiol in human breast cyst fluid. *Eur. J. Cancer*, **26**, 481–484.

LEIS HP. (1993). Gross Breast Cysts: significance of apocrine type, identification by cyst fluid analysis and management. *Breast Disease*, **6**, 185–194.

MILLER WR, SCOTT WN, HARRIS WH AND WANG D. (1992). Using biological measurements, can patients with benign breast disease who are at high risk for breast cancer be identified. *Cancer Detect. Prev.*, **16**, 99–106.

NALDONI C, COSTANTINI M, DOGLIOTTI L, BRUZZI P, BUCCHI L, BUZZI G, TORTA M AND ANGELI A. (1992). Association of cyst type with risk factors for breast cancer and relapse rate in women with gross cystic disease of the breast. *Cancer Res.*, **52**, 1791–1795.

NESS JC, SEDGHINASAB M, MOE RE AND TAPPER D. (1993). Identification of multiple proliferative growth factors in breast cyst fluid. *Am. J. Surg.*, **166**, 237–243.

ONDETTI MA, WILLIAMS NJ, SABO EF, PLUSCEC J, WEAVER ER AND KOCY O. (1971). Angiotensin-Converting Enzyme Inhibitors from the venom of Bothrops jararaca. Isolation, elucidation of structure and synthesis. *Biochemistry*, **19**, 4033–4039.

PAGE DL, VAN DER ZWAAG R, ROGERS LW, WILLIAMS LT, WALKER WE AND HARTMANN WH. (1978). Relation between component parts of fibrocystic disease complex and breast cancer. *J. Natl Cancer Inst.*, **61**, 1055–1063.

PARISH DC. (1994). The role of proteolysis in tumour invasion and growth. *Endocrine-Related Cancer*, **1**, 19–36.

PEKONEN F, NYMAN T, ÄMMÄLÄ M AND RUTANEN E-M. (1995). Decreased expression of messenger RNAs encoding endothelin receptors and neutral endopeptidase 24.11 in endometrial cancer *Br. J. Cancer*, **71**, 59–63.

REED MJ, ANGELI A, THIJSSEN JH, MILEWICZ A, SZAMEL L, HUBER J, HARLOZINSKA-SZMYRKA A, TOTH J AND KORNAFEL J. (1994). Gross cystic breast disease: overview and directions for future research. *Endocrine-Related Cancer*, **2**, 9–13.

SANCHEZ LM, VIZOSO F, ALLENDE MT, RUIBAL A AND LOPEZ-OTIN C. (1992). Quantification and molecular analysis of Cathepsin D in breast cyst fluids. *Eur. J. Cancer*, **28A**, 828–832.

SCAMBIA G, PANICI PD, FERRANDINA G, BATTAGLIA F, ROSSI S, BELLANTONE R, CRUCITTI F AND MANCUSO S. (1991). Cathepsin D and epidermal growth factor in human breast cyst fluid. *Br. J. Cancer*, **64**, 965–967.

SCHON HJ, SCHURZ B, WENZL R, KNOGLER W AND KUBISTA E. (1993). $\beta$-Endorphin, steroids and prolactin immunoassay in breast cysts and blood. *Arch. Pathol. Lab. Med.*, **117**, 248–253.

SCHREY MP AND PATEL KV. (1994). Role of regulatory peptides in the control of breast cancer cell growth and function. *Endocrine-Related Cancer*, **3**, 41–70.

SCHURZ B, SCHON HJ, WENZL R, KUBISTA E, SPONA J, HUBA J AND WEINDLMAYR-GOETTEL M. (1991). Breast cyst fluid concentrations of beta-endorphin, steroids and gonadotrophins in premenopausal women with gross cystic disease. *Maturitas*, **13**, 123–128.

SCHWEISFURTH H, SCHMIDT M, BRUGGER E, MAIWALD L AND THIEL H. (1985) Alterations of serum carboxypeptidases N and angiotensin-I converting enzyme in malignant diseases. *Clin. Biochem.*, **18**, 242–246.

SETHI T AND ROZENGURT E. (1991). Multiple neuropeptides stimulate clonal growth of small cell lung cancer: effects of bradykinin, vasopressin, cholecystokinin, galanin and neurotensin. *Cancer Res.*, **51**, 3621–3623.

SHIPP MA, VIJARAGHAVAN J, SCHMIDT E, MASTELLER EL, D'ADAMIO L, HERSH LB AND REINHERZ ELA. (1989). Common acute lymphoblastic leukemia antigen (CALLA) is active neutral endopeptidase 24.11 ('enkephalinase'): direct evidence by cDNA transfection analysis. *Proc. Natl Acad. Sci USA*, **86**, 297–301.

SHIPP MA, TARR GE, CHENN C-Y, SWITZER SN, HERSCH LB, STEIN H, SUNDAY ME AND REINHERZ EL. (1991). CD10/neutral endopeptidase 24.11 hydrolyzes bombesin-like peptides and regulates the growth of small cell carcinoma of the lung. *Proc. Natl Acad. Sci. USA*, **88**, 10662–10666.

SPILLANTINI MG, SICUTERI F, SALMON S AND MALFROY B. (1990). Characterisation of Endopeptidase 3.4.24.11 (Enkephalinase) activity in human plasma and cerebrospinal fluid. *Biochem. Pharmacol.*, **39**, 1353–1356.

VARELA SA AND SAEZ JJBL. (1993). Utility of serum activity of angiotensin-converting enzyme as a tumour marker. *Oncology*, **50**, 430–435.

WEBER CJ, O'DORISIO TM, HOWE B, KOSCHITZKY T AND MERRIAM L. (1989). Gastrin releasing peptide-, calcitonin gene-related peptide-, and calcitonin-like immunoreactivity in human breast cyst fluid and gastrin releasing peptide-like immunoreactivity in human breast carcinoma cell lines. *Surgery*, **106**, 1134–1140.